# Salty Food Preference and Intake and Risk of Gastric Cancer: The JACC Study

**DOI:** 10.2188/jea.JE20150023

**Published:** 2016-02-05

**Authors:** Mitsumasa Umesawa, Hiroyasu Iso, Yoshihisa Fujino, Shogo Kikuchi, Akiko Tamakoshi

**Affiliations:** 1 Department of Public Health, Dokkyo Medical University School of Medicine, Mibu, Tochigi, Japan; 2 Department of Public Health Medicine, Faculty of Medicine, University of Tsukuba, Tsukuba, Ibaraki, Japan; 3 Public Health, Department of Social and Environmental Medicine, Graduate School of Medicine, Osaka University, Suita, Osaka, Japan; 4 Department of Preventive Medicine and Community Health, School of Medicine, University of Occupational and Environmental Health, Kitakyushu, Fukuoka, Japan; 5 Department of Public Health, Aichi Medical University, Nagakute, Aichi, Japan; 6 Department of Public Health, Hokkaido University Graduate School of Medicine, Sapporo, Japan

**Keywords:** salt, gastric cancer, prospective study

## Abstract

**Background:**

High sodium intake is a potential risk factor of gastric cancer. However, limited information is available on the relationship between salty food preference or intake and risk of gastric cancer. The aim of the present study was to determine the association between these variables among the Japanese population.

**Methods:**

Between 1988 and 1990, 15 732 men and 24 997 women aged 40–79 years old with no history of cancer or cardiovascular disease completed a lifestyle questionnaire that included information about food intake. The subjects were enrolled in the Japan Collaborative Cohort (JACC) Study for Evaluation of Cancer Risk Sponsored by Monbusho. After a median follow-up of 14.3 years, 787 incident gastric cancers were documented. We examined the associations between salty food preference and intake and gastric cancer incidence using the Cox proportional hazard model.

**Results:**

The risk of gastric cancer among subjects with a strong preference for salty food was approximately 30% higher than among those who preferred normal-level salty food (hazard ratio [HR] 1.31; 95% confidence interval [CI], 1.02–1.67). The risk of gastric cancer in subjects who consumed 3 and ≥4 bowls/day of miso soup was approximately 60% higher than in those who consumed less miso soup (HR 1.67; 95% CI, 1.16–2.39 and HR 1.64; 95% CI, 1.11–2.42, respectively). Sodium intake correlated positively and linearly with risk of gastric cancer (*P* for trend = 0.002).

**Conclusions:**

The present study showed that salty food preference, consumption of large quantities of miso soup, and high sodium intake were associated with increased risk of gastric cancer among Japanese people.

## INTRODUCTION

Gastric cancer is one of the main causes of cancer deaths. According to the World Health Organization, gastric cancer is the second-leading cause of cancer deaths, with 736 000 gastric cancer-related deaths worldwide in 2008. Another study showed that the incidence of gastric cancer in Eastern Asia is the highest in the world; the incidence rates in Eastern Asia were 42.4/100 000 in men and 18.3/100 000 in women, which were over four times higher than in Western Europe and over 7 times higher than in North America.^1^

Excess salt intake is an important risk factor of gastric cancer. Several epidemiological studies showed a positive association between salt intake and risk of gastric cancer.^2^^,^^3^ The association between salty food intake and risk of gastric cancer has also been examined in several epidemiological studies.^3^^–^^7^ The results of these studies suggest that salty preserved animal food intake correlates with risk of gastric cancer; however, findings on the association between the intake of salty preserved plant foods (eg, miso [soybean-paste] soup) and risk of gastric cancer have been inconsistent. In addition, recent Asian epidemiological studies showed that salty taste preference was positively associated with risk of gastric cancer.^8^^,^^9^ However, the evidence is limited in Japanese people.

The aim of the present study was to determine the correlation between salty food preference/salty food intake and risk of gastric cancer. We tested our a priori hypothesis that both salty food preference and salty food intake are associated with risk of gastric cancer using data of a large prospective cohort study of middle-aged Japanese people from the general population.

## MATERIALS AND METHODS

### Subjects

The Japan Collaborative Cohort (JACC) Study for Evaluation of Cancer Risks, sponsored by the Ministry of Education, Culture, Sports, and Science, was conducted from 1988 to 1990. A total of 110 585 subjects (46 395 men and 64 190 women) aged 40 to 79 years completed self-administered questionnaires about their lifestyles and medical histories on cancer and cardiovascular disease. The population sample was recruited from 45 communities across Japan, and most of the participants completed questionnaires and municipal health screening examinations according to the Health Law for the Aged in their communities.^10^ The sampling methods and protocols of the JACC Study have been described in detail previously.^10^^,^^11^ We excluded from the present analysis 5850 subjects (2574 men and 3276 women) with medical history of cancer, stroke, and coronary heart disease at baseline. Of the remaining 104 735 subjects, 58 645 subjects (23 099 men and 35 546 women) provided valid responses to the dietary intake-related questions. Of these, we used the data of 40 729 subjects (15 732 men and 24 997 women) who lived in 24 communities and underwent follow-up research on cancer incidence to examine the association between salty food preference and salty food intake and risk of gastric cancer.

### Follow-up

The median follow-up period was 14.3 years. The number of subjects who moved out of the study area was 1921 (4.7%). The incidence of cancer was mainly based on the records of population-based cancer registries and systematic review of death certificates. In some study areas, the medical records of hospitals were also reviewed. The incidence data were coded according to the 10th Revision of the International Statistical Classification of Diseases and Related Health Problems (ICD-10). We defined gastric cancer as C16.0 to C16.9. The present study was approved by the ethics committee of Hokkaido University.

### Dietary intake, nutrition intake, and salty taste preference

The questionnaire included items on salty taste preference, with five potential responses: “not favored at all”, “slightly not favored”, “no opinion”, “slightly favored”, and “strongly favored”. Each participant was asked to record the frequency of intake of 35 foods. Five responses were possible for 33 of the food items: “rarely”, “1 to 2 days per month”, “1 to 2 days per week”, “3 to 4 days per week”, and “almost every day”. Four responses were possible for miso (salty soybean paste) soup intake: “rarely”, “a few times per month”, “almost every other day”, and “every day”. Those who chose “everyday” were asked about the number of servings (bowls) per day. Rice intake was quantified as bowls (140 g) per day.

The reproducibility and validity of this dietary questionnaire have been reported elsewhere.^12^ The average daily intake of nutrients and total energy was calculated by multiplying the frequency of consumption of each item by its nutrient content and energy per serving and totaling the nutrient intake for all food items. The energy-adjusted nutrient intake was calculated by the residual method.

### Statistical analysis

Statistical analysis was based on age- and sex-adjusted incidence rates of gastric cancer during the follow-up period from 1989 to 2009. For each participant, the person-years of follow-up were calculated from the date of filling out the baseline questionnaire to the incidence of gastric cancer, death, moving out of the community, or the end of the follow-up, whichever occurred first. Age- and sex-adjusted and multivariate-adjusted hazard ratios (HRs) of gastric cancer were defined as the incidence rate according to the salty food preference and the frequency of salty food intake. The HRs of gastric cancer and their 95% confidence intervals (CIs) were calculated after adjustment for age, sex, and potential confounding factors using the Cox proportional hazard model. We analyzed the HR according to salty food preference using subjects who selected “no opinion” as the answer to their salty food preference for the reference group. We also tested for a linear trend across the categories of salty food preference by assigning 1 to 5 to the corresponding category. We defined salty food as miso (salty soybean paste) soup, ham/sausage, fish paste, dried and salted fish, salty pickles, preserved foods using soy sauce, and boiled beans. We used subjects who rarely consumed each of the foods (except for miso soup) as the reference group. Since subjects who never consume miso soup are rare among Japanese people, the reference group comprised subjects who consumed miso soup only a few times per week. Furthermore, we analyzed the HR according to sex-specific quintiles of sodium intake: the median values of the lowest to highest quintiles of sodium intake were 1052, 1572, 1951, 2376, and 3017 mg/day, respectively. We used the lowest quintile as a reference. We tested sex interaction in each analysis and found no significant interactions. Thus, we present the data of the entire study population rather than separate data for men and women.

The mean values and proportions of gastric cancer risk factors are presented according to salty food preference and frequency of salty food intake. Age- and sex-adjusted and multivariate-adjusted HRs and their 95% CIs were calculated after adjustment for age, sex, and potential confounding factors, including family history of gastric cancer, body mass index (BMI; kg/m^2^), alcohol intake (current drinker, not current drinker, and missing information), smoking status (never, ex-smoker, current smoker, and missing information), walking time/day (never, about 30 min, 30–60 min, ≥60 min/day, and missing information), educational status (education until 12, 13 to 15, 16 to 18, or ≥19 years of age, and missing information) and perceived mental stress (low, medium, high, extremely high, and missing information). We also used the frequency of total vegetable intake (sex-specific quintiles of sum of frequency of consumption of green leafy vegetables, carrot or pumpkin, tomatoes, cabbage or head lettuce, and Chinese cabbage and missing information) and fruit intake (sex-specific quintiles of sum of frequency of consumption of citrus fruits and other fruits and missing information) as confounding variables. As for salty food preference, we calculated multivariate-adjusted HRs and their 95% CIs with adjustment for sodium intake (sex-specific quintiles and missing information).

All analyses were conducted using SAS version 9.3 software (SAS Institute Inc., Cary, NC, USA). A *P* value less than 0.05 denoted the presence of statistical significance, and those of 0.05–0.10 were regarded as borderline significance.

## RESULTS

Table 1 summarizes the age- and sex-adjusted characteristics of subjects based on salty food preference. Of 40 729 subjects, 37 672 responded to the question regarding salty taste preference. Salty food preference correlated positively with gender (men), BMI, current alcohol drinking, and current smoking and negatively with age and frequency of vegetable and fruit intake, but did not correlate with family history of gastric cancer. In addition, the estimated sodium intake was higher in subjects who preferred salty food than in those who did not.

**Table 1.** Age- and sex-adjusted characteristics of the subjects according to preference for salty food

	Preference of salty food					*P* for difference

	not favored at all	slightly not favored	no opinion	slightly favored	strongly favored	
*n*	813	5243	18 958	9484	3174	
Age, years	57.9	56.3	56.4	55.2	54.4	<0.001
Men, %	32	27	33	50	55	<0.001
Body mass index, kg/m^2^	22.5	22.5	22.7	22.9	23.0	<0.001
Current drinker, %	39	41	43	48	51	<0.001
Current smoker, %	20	21	22	26	32	<0.001
Family history of gastric cancer, %	12.2	14.1	13.5	13.2	14.3	0.258
Frequency of total vegetable intake, servings/week	17.8	16.3	15.7	15.3	15.6	<0.001
Frequency of fruit intake, servings/week	8.2	8.0	7.9	7.8	7.9	0.002
Mean sodium intake, mg/day	1828	1827	1980	1992	2037	<0.001

Table 2 lists the age- and sex-adjusted characteristics of subjects based on the frequency of miso soup intake. Of 40 729 subjects, 38 763 responded to the question regarding miso soup intake. The frequency of miso soup intake correlated positively with gender (men), frequency of total vegetable intake, and sodium intake and negatively with current smoking. Subjects who drank miso soup almost every other day or 1–2 bowls per day tended to be younger, have lower mean BMI, and be less likely to have a family history of gastric cancer compared with the other subjects.

**Table 2.** Age- and sex-adjusted characteristics of the subjects according to frequency of miso soup intake

	Frequency of miso soup intake							

	Never	A few times per month	Almost every second day	Everyday				*P* for difference

				1 bowl per day	2 bowls per day	3 bowls per day	4 bowls or more per day	
*n*	2160	3764	5655	6979	8669	8476	3060	
Age, years	56.3	56.4	54.2	54.5	54.8	58.1	60.7	<0.001
Men, %	29	34	33	32	41	43	60	<0.001
Body mass index, kg/m^2^	22.8	22.8	22.7	22.6	22.7	22.9	23.1	<0.001
Current drinker, %	44	48	45	44	44	42	45	<0.001
Current smoker, %	26	25	25	23	23	24	23	<0.001
Family history of gastric cancer, %	13.6	13.6	13.3	13.3	12.9	13.9	14.1	0.029
Frequency of total vegetable intake, servings/week	13.3	13.4	14.8	16.2	16.3	17.0	17.0	<0.001
Frequency of fruit intake, servings/week	7.4	7.2	7.6	7.6	8.1	8.2	7.2	<0.001
Mean sodium intake, mg/day	1136	1187	1421	1808	2176	2562	2818	<0.001

Table 3 shows the age- and sex-adjusted characteristics of subjects based on sex-specific quintiles of sodium intake. Sodium intake correlated positively with age, frequency of vegetable intake, and fruit and miso soup intake but negatively with current drinking and current smoking. Subjects in the middle quintile of sodium intake had lower BMI compared to those with the highest and lowest intake.

**Table 3.** Age- and sex-adjusted characteristics of the subjects according to sex-specific quintiles of sodium intake

	Quintiles of sodium intake					*P* for difference

	1 (lowest)	2	3	4	5 (highest)	
*n*	8145	8147	8145	8147	8145	
Median sodium intake, mg/day	1052	1572	1951	2376	3017	
Age, years	54.8	55.7	55.9	56.4	57.9	<0.001
Men, %	39	39	39	39	39	—
Body mass index, kg/m^2^	22.9	22.7	22.7	22.8	22.9	<0.001
Current drinker, %	49	47	44	43	39	<0.001
Current smoker, %	26	23	24	24	23	<0.001
Family history of gastric cancer, %	13.4	13.2	13.5	14.0	13.1	0.466
Frequency of total vegetable intake, servings/week	13.3	15.1	16.2	16.6	17.9	<0.001
Frequency of fruit intake, servings/week	6.9	7.7	8.0	8.2	8.2	<0.001
Frequency of miso soup intake, servings/week	3.2	7.2	11.6	16.6	20.7	<0.001

During the median 14.3-year follow-up period of the 40 729 subjects, 787 incident cases of gastric cancer were documented. Table 4 presents the age- and sex-adjusted and multivariate-adjusted HRs (and 95% CIs) and *P* value for the linear trend in gastric cancer risk across the categories according to salty food preference. The risk of gastric cancer was significantly higher among subjects who considered their salty food preference “strongly favored” compared with subjects who considered it “no opinion”, even after adjustment for potential confounding factors, such as vegetable and fruit intake (HR 1.31; 95% CI, 1.03–1.68). The association did not change even after further adjustment for sodium intake (HR 1.31; 95% CI, 1.02–1.67).

**Table 4.** Age- and sex-adjusted and multivariable-adjusted hazard ratios of gastric cancer according to preference of salty food

	Preference of salty food					*P* for trend

	not favored at all	slightly not favored	no opinion	slightly favored	strongly favored	
*n*	813	5243	18 958	9484	3174	
Person-years	9971	66 249	247 940	125 428	42 534	
Number of incidents	13	82	333	193	82	
Number of incidents per 1000 person years	1.30	1.24	1.34	1.54	1.93	
Age- and sex-adjusted HR (95% CI)	0.87 (0.50–1.51)	0.92 (0.72–1.16)	1.00	1.02 (0.86–1.21)	1.30 (1.03–1.66)	0.020
Multivariable-adjusted HR (95% CI)^a^	0.92 (0.53–1.61)	0.97 (0.76–1.23)	1.00	1.05 (0.88–1.25)	1.32 (1.04–1.69)	0.038
Multivariable-adjusted HR (95% CI)^b^	0.91 (0.52–1.58)	0.96 (0.76–1.22)	1.00	1.05 (0.88–1.26)	1.31 (1.03–1.68)	0.035
Multivariable-adjusted HR (95% CI)^c^	0.94 (0.54–1.63)	0.99 (0.78–1.26)	1.00	1.05 (0.88–1.25)	1.31 (1.02–1.67)	0.059

CI, confidence interval; HR, hazard ratio.

^a^Adjusted further for body mass index (kg/m^2^), ethanol intake (current drinker or not and missing), smoking status (four categories), family history of gastric cancer, walking time (five categories), educational status (five categories), and perceived mental stress (four categories).

^b^Adjusted further for frequency of total vegetable intake (sex-specific quintiles and missing) and fruit intake (sex-specific quintiles and missing).

^c^Adjusted further for sodium intake (sex-specific quintiles and missing).

Table 5 shows the HRs (and 95% CIs) of gastric cancer according to the frequency of miso soup intake. Compared with subjects who took miso soup a few times per month, the risk of gastric cancer was significantly higher among those who drank more than 4 bowls per day (HR 1.64; 95% CI, 1.11–2.42) and subjects who took 3 bowls per day (HR 1.67; 95% CI, 1.16–2.39). The risk of gastric cancer also tended to be higher among subjects who took 2 bowls of miso soup per day than in the reference group, though with only borderline statistical significance (HR 1.42; 95% CI, 0.98–2.04, *P* = 0.063).

**Table 5.** Age- and sex-adjusted and multivariable-adjusted hazard ratios of gastric cancer according to frequency of miso soup intake

	Never	A few times per month	Almost every second day	Everyday			

				1 bowl per day	2 bowls per day	3 bowls per day	4 bowls or more per day
*n*	2160	3764	5655	6979	8669	8476	3060
Person-years	23 927	36 670	66 626	83 550	126 481	127 919	44 163
Number of incidents	28	35	69	81	171	249	112
Number of incidents per 1000 person years	1.17	0.95	1.04	0.97	1.35	1.95	2.54
Age- and sex-adjusted HR (95% CI)	1.31 (0.80–2.16)	1.00	1.21 (0.80–1.82)	1.13 (0.76–1.67)	1.44 (1.00–2.07)	1.71 (1.20–2.43)	1.70 (1.16–2.49)
Multivariable-adjusted HR (95% CI)^a^	1.30 (0.79–2.14)	1.00	1.21 (0.81–1.82)	1.13 (0.76–1.67)	1.43 (0.99–2.06)	1.68 (1.18–2.40)	1.66 (1.13–2.45)
Multivariable-adjusted HR (95% CI)^b^	1.30 (0.79–2.14)	1.00	1.21 (0.81–1.82)	1.11 (0.75–1.66)	1.42 (0.98–2.04)	1.67 (1.16–2.39)	1.64 (1.11–2.42)

CI, confidence interval; HR, hazard ratio.

^a^Adjusted further for body mass index (kg/m^2^), ethanol intake (current drinker or not and missing), smoking status (four categories), family history of gastric cancer, walking time (five categories), educational status (five categories), and perceived mental stress (four categories).

^b^Adjusted further for frequency of total vegetable intake (sex-specific quintiles and missing) and fruit intake (sex-specific quintiles and missing).

Table 6 shows the HRs and 95% CIs of gastric cancer according to quintiles of sodium intake. The risk of gastric cancer was higher in subjects with the highest and middle quintiles of sodium intake compared to those of the lowest quintile (high sodium intake: HR 1.51; 95% CI, 1.17–1.94, middle sodium intake: HR 1.36; 95% CI, 1.05–1.76, *P* for trend = 0.002). With regard to subjects of the second highest quintile of sodium intake (the fourth quintile), the risk of gastric cancer tended to be significant, though with borderline statistical significance (HR 1.29; 95% CI, 1.00–1.67, *P* = 0.051).

**Table 6.** Associations between sex-specific quintiles of sodium intake and risk of gastric cancer

	Quintiles of sodium intake					*P* for trend

	1 (low)	2	3	4	5 (high)	
*n*	8145	8147	8145	8147	8145	
Person-years	90 603	96 858	107 271	118 984	121 564	
Number of incidents	92	127	161	178	229	
Number of incidents per 1000 person years	1.02	1.31	1.50	1.50	1.88	
Age- and sex-adjusted HR (95% CI)	1.00	1.20 (0.92–1.57)	1.36 (1.05–1.76)	1.31 (1.02–1.69)	1.51 (1.18–1.93)	<0.001
Multivariable-adjusted HR (95% CI)^a^	1.00	1.21 (0.92–1.58)	1.37 (1.06–1.77)	1.30 (1.01–1.68)	1.53 (1.19–1.96)	<0.001
Multivariable-adjusted HR (95% CI)^b^	1.00	1.20 (0.92–1.58)	1.36 (1.05–1.76)	1.29 (1.00–1.67)	1.51 (1.17–1.94)	0.002

CI, confidence interval; HR, hazard ratio.

^a^Adjusted further for body mass index (kg/m^2^), ethanol intake (current drinker or not and missing), smoking status (four categories), family history of gastric cancer, walking time (five categories), educational status (five categories), and perceived mental stress (four categories).

^b^Adjusted further for frequency of total vegetable intake (sex-specific quintiles and missing) and fruit intake (sex-specific quintiles and missing).

Finally, we examined the associations between the frequency of salty food intake (excluding miso soup) and risk of gastric cancer. The risk of gastric cancer did not correlate with consumption of ham/sausage, fish paste, dried and salted fish, salty pickles, or boiled beans (data not shown). The risk of gastric cancer did tend to be higher among subjects who consumed foods preserved using soy sauce almost every day compared to those who consumed these products rarely; however, the association was of borderline statistical significance (HR 1.28; 95% CI, 0.97–1.71, *P* = 0.087).

## DISCUSSION

The main findings of the present study were that strong salty food preference was associated with approximately 30% higher risk of gastric cancer than subjects who preferred normal-level salty food, and miso soup intake of 3 or more bowls per day was associated with approximately 60% higher risk of gastric cancer than subjects who took miso soup a few times per month. The estimated sodium intake itself also correlated with risk of gastric cancer.

Our findings in Japanese individuals add support to the findings of previous studies from other Asian countries. A prospective study of Korean men and women reported that the risk of gastric cancer was 10% higher in subjects who preferred salty foods compared to those who did not.^8^ A cross-sectional study of Chinese subjects showed that patients with gastric cancer preferred salty taste compared with healthy individuals.^9^ With regard to the consumption of high-salt foods, a prospective study of Japanese subjects showed that the risk of gastric cancer was about 80% to 100% higher in subjects who took miso soup every day compared with subjects who did not.^3^ Another prospective study of Japanese subjects showed a positive association between miso soup intake and risk of gastric cancer, although the association was not statistically significant.^6^ Several epidemiological studies showed a positive association between salty pickle intake and risk of gastric cancer.^3^^,^^4^^,^^6^ However, in the present study, there was no association between salty pickle intake and risk of gastric cancer. As for sodium intake, two prospective studies of Japanese subjects showed a positive association between high sodium intake and risk of gastric cancer,^2^^,^^3^ which is similar to the finding of the present study. In addition, we found a significant association between salty food preference and risk of gastric cancer even after adjustment for sodium intake. The mechanisms for that association may include tissue damage caused by high concentrations of sodium in the diet^13^ and carcinogenesis by N-Methyl-N′-nitro-N-nitrosoguanidine enhanced by high concentrations of sodium in the diet.^14^

The strength of the present study was its community-based prospective cohort design and inclusion of a large free-living population. In addition, the questionnaire included direct and detailed questions about salty food preference, which allowed the exact relationship between salty food preference and risk of gastric cancer in the Japanese subjects to be defined.

The main limitation of the present study relates to the effect of *Helicobacter pylori* infection. This issue was not investigated in this study due to lack of information for all subjects on such infection. High sodium intake is reported to weaken the protective effect of the mucous barrier and promote the carcinogenic effect of *H. pylori* infection,^15^^,^^16^ a recognized risk factor of gastric cancer. Therefore, we also examined the relationship between salty food preference/salty food intake and risk of gastric cancer based on information on *H. pylori* infection, which was measured in a case-control study,^17^ although the number of subjects included in this analysis was limited (*n* = 579). Adjustment for *H. pylori* infection did not alter the association between salty food preference/salty food intake and risk of gastric cancer (data not shown).

Several mechanisms may account for the association between salty food intake and risk of gastric cancer. First, a high concentration of sodium in the stomach is reported to cause acute damage of the surface mucosal cells and transient cell proliferation.^13^ Second, a salty diet alters mucin production and enhances gastric chemical carcinogenesis.^18^ Both mechanisms are salt dose-dependent. In addition, the relationship between high sodium intake and *H. pylori* infection stated above may enhance the development of gastric cancer.

In conclusion, our large prospective study of Japanese men and women showed that salty food preference correlated positively with risk of gastric cancer. Intake of large quantities of miso soup was also associated with risk of gastric cancer. These findings suggest that education about the potential harmful effects of salty food preference and reduction of sodium intake (especially miso soup) may help prevent the development of gastric cancer among Japanese people.
